# Role of eotaxin-1/CCL11 in sepsis-induced myocardial injury in elderly patients

**DOI:** 10.18632/aging.102896

**Published:** 2020-03-09

**Authors:** Ying Li, Youguang Zhao, Chenming Qiu, Yuanrui Yang, Guihua Liao, Xi Wu, Xiaowan Zhang, Qian Zhang, Ru Zhang, Zhang Wang

**Affiliations:** 1 Department of Geriatrics, The General Hospital of Western Theater Command, Chengdu 610083, Sichuan, China; 2 Department of Urology, The General Hospital of Western Theater Command, Chengdu 610083, Sichuan, China; 3 Department of Cardiology, The General Hospital of Western Theater Command, Chengdu 610083, Sichuan, China

**Keywords:** septic cardiomyopathy, sepsis, CCL11, eotaxin-1, biomarker

## Abstract

Myocardial injury is a serious complication of sepsis. The present study aimed to identify potential biomarkers of sepsis-induced myocardial injury. Differentially expressed genes (DEGs) in patients and mice with sepsis-induced myocardial injury were identified via bioinformatic analysis. The identified DEG was tested in elderly patients with sepsis-induced myocardial injury. We identified 19 co-expressed DEGs. The most significant DEG was eotaxin-1/CCL11. We enrolled 25 controls without infections and 28 patients with sepsis-induced myocardial injury. Six of patients died within 30 days. Circulating eotaxin-1/CCL11 levels were significantly higher in patients with sepsis-induced myocardial injury than controls and were higher in non-survivors than survivors (both *P* < 0.01). Eotaxin-1/CCL11 was positively correlated with troponin I (r=0.48, *P*=0.01), B-type natriuretic peptide (BNP, r=0.44, *P*=0.02), and white blood cell (WBC) count (r=0.41, *P*=0.03). For the prediction of 30-day mortality, eotaxin-1/CCL11 had the greatest discriminatory ability (AUC 0.97) compared with troponin I (AUC 0.89), BNP (AUC 0.80), and WBC count (AUC 0.86). Taken together, eotaxin-1/CCL11 was upregulated in sepsis-injured myocardium and circulating eotaxin-1/CCL11 was a biomarker for predicting severity and mortality of elderly patients with sepsis-induced myocardial injury. These results suggest that eotaxin-1/CCL11 may become a useful biomarkers and potential therapeutic target for sepsis-induced myocardial injury.

## INTRODUCTION

Sepsis is considered as a life-threatening condition caused by dysregulated, inappropriate host response to an infection, such as pneumonia. Particularly in elderly patients, sepsis remains a leading cause of death worldwide [1]. Myocardial dysfunction or cardiomyocyte injury frequently occurs in patients with sepsis, which is known as septic cardiomyopathy, an emerging challenge in clinical practice [2, 3]. Sepsis-induced myocardial injury, characterized by elevation in biomarkers of cardiomyocyte injury, is an important contributor to multiorgan dysfunction in sepsis and associated with increased morbidity and mortality [4–6]. Currently, treatment of sepsis-induced myocardial injury mainly focuses on treating the underlying infection and sepsis and providing nonspecific supportive care [5]. In fact, there is no specific therapy for sepsis-induced myocardial injury owing to incomplete understanding of the underlying pathogenesis.

Microarray analysis in transcriptome profile has been used as a valuable laboratory tool to screen critical genes and signaling pathways involved in pathogenesis and to identify novel therapeutic targets for treatment. Transcriptome profiling usually yields a considerable number of potential differential transcripts, and it is a challenge to identify the critical genes. A pooled biostatistical analysis of transcriptome array datasets which combines multiple species across the animal kingdom may help screen conservative and important genes involved in a given disorder [7]. In the present study, we aimed to identify key genes and pathways which critically contribute to the development and progression of sepsis-induced myocardial injury through analyzing the common differential genes in both humans and mice with sepsis-induced myocardial injury. The potential role of the common critical gene candidate was then tested in elderly patients with sepsis-induced myocardial injury.

Elderly patients (≥80 years of age) have a high prevalence of pre-existing cardiovascular diseases, especially congestive heart failure [8]. Infection is a significant cause of death in elderly patients with acutely decompensated heart failure [9, 10]. Sepsis-related myocardial injury may contribute to the high mortality in elderly patients with sepsis [10]. Unfortunately, clinical characteristics of elderly patients with sepsis-induced myocardial injury were not well documented in previous studies. In the present study, we analyzed the correlation between the circulating levels of the most significantly differential gene identified in biostatistical analysis and the severity and mortality of sepsis-induced myocardial injury in elderly patients.

## RESULTS

### Identification of DEGs

**Figure 1:**
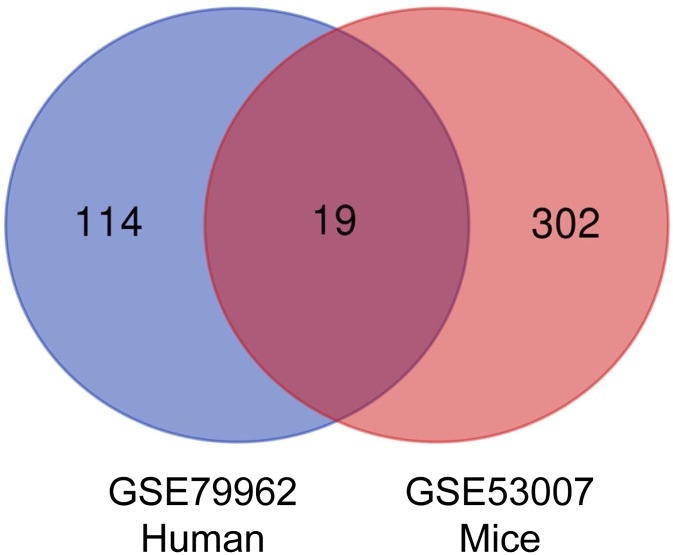
**Venn diagram of DEGs.** Intersection analysis between DEGs in GSE79962 and GSE53007 microarray datasets. The 19 overlapped genes were related to sepsis-induced myocardial injury. DEGs, differentially expressed genes.

**Figure 2:**
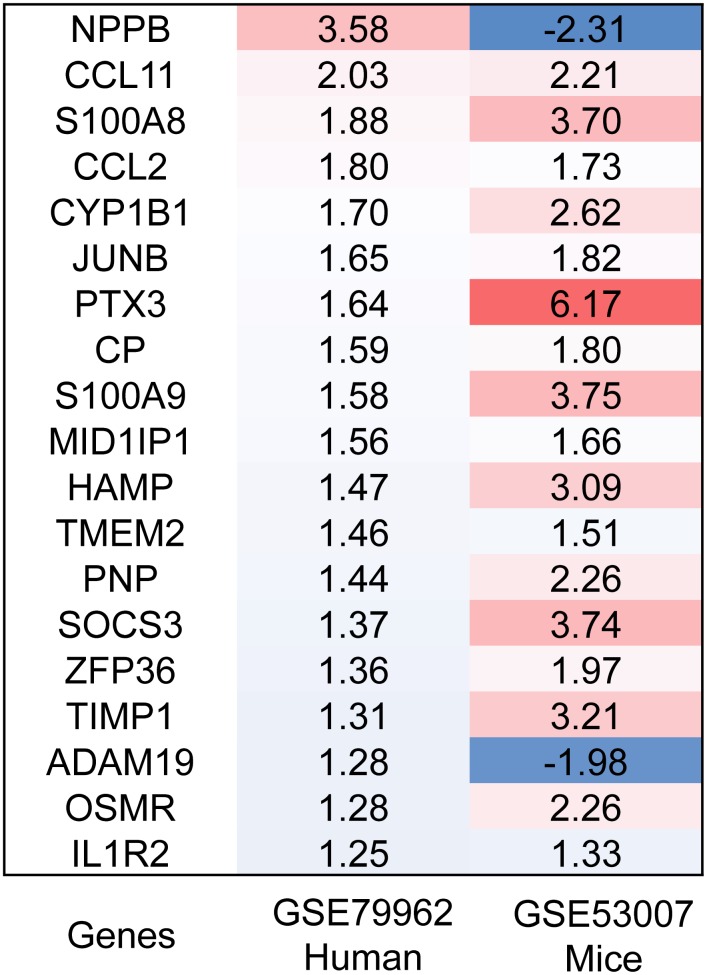
**Heatmap of DEGs.** The expression levels of the 19 overlapped genes in human and mouse sepsis-injured myocardium. DEGs, differentially expressed genes. Red, greater expression. Blue, less expression.

We found 133 differentially expressed genes (DEGs) in human heart specimens of sepsis-induced myocardial injury patients compared with nonfailing human heart specimens, including 39 down-regulated genes and 94 up-regulated genes. In addition, we found 321 DEGs in heart specimens from mice with sepsis-induced myocardial injury compared with control mice, including 85 down-regulated genes and 236 up-regulated genes. We defined 19 common DEGs (Co-DEGs) in human and mouse sepsis-injured myocardium (Figure 1). The only gene with log 2-fold-change (logFC) greater than 2 in both human and mice was C-C motif chemokine 11 (CCL11), also known as eotaxin-1 (Figure 2).

### Functional enrichment and ppi network analysis in Co-DEGs

**Figure 3:**
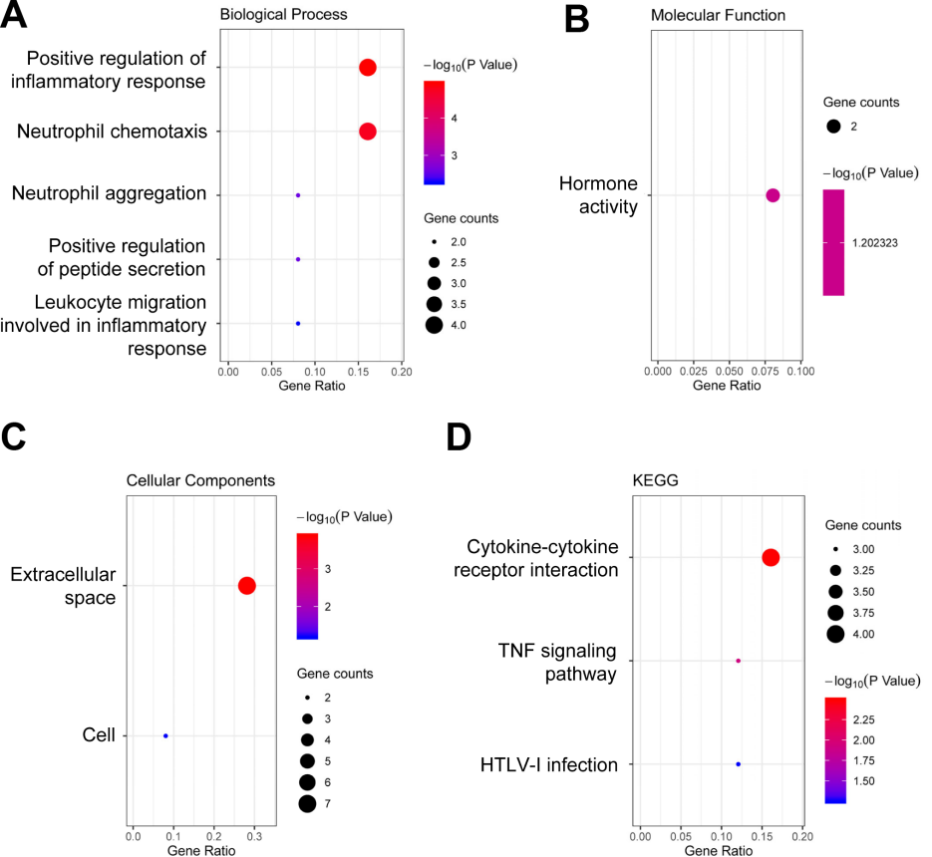
**Functional enrichment analysis of DEGs.** (**A**) GO analysis of biological process. (**B**) GO analysis of molecular function. (**C**) GO analysis of cellular components. (**D**) KEGG pathway enrichment analysis. DEGs, differentially expressed genes. Dot sizes represent counts of enriched DEGs, and dot colors represent negative Log10-P values.

**Figure 4:**
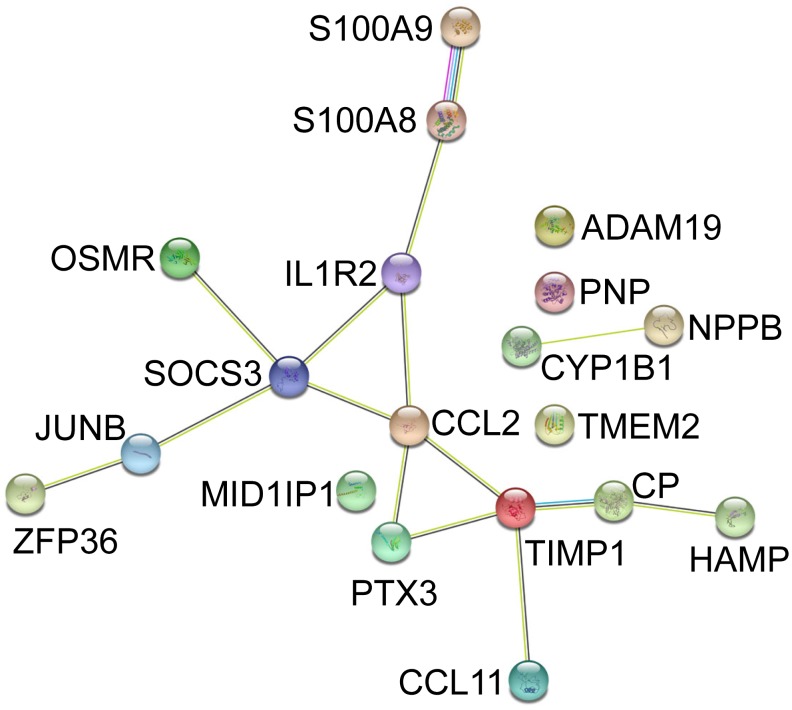
**PPI of Co-DEGs in GSE79962 and GSE53007 constructed by STRING database.** PPI, protein-protein interaction; DEGs, differentially expressed genes.

Gene ontology (GO) enrichment analysis of the 19 Co-DEGs was performed using DAVID. As presented in Figure 3, the results showed that the overrepresented GO terms in biological processes were enriched in positive regulation of inflammatory response, neutrophil chemotaxis, neutrophil aggregation, positive regulation of peptide secretion, and leukocyte migration involved in inflammatory response (Figure 3A). The enriched GO term in molecular function was hormone activity (Figure 3B). The enriched GO terms in cellular components were extracellular space and cell (Figure 3C). Kyoto encyclopedia of genes and genomes (KEGG) pathway analysis results indicated that the Co-DEGs were mainly enriched in pathways of cytokine-cytokine receptor interaction, tumor necrosis factor (TNF) signaling pathway, and human T-lymphotropic virus type 1 (HTLV-I) infection (Figure 3D). In the PPI network, there were 19 nodes (proteins) and 15 edges (interactions; Figure 4). The hub notes were C-C motif chemokine 2 (CCL2), tissue inhibitor of metalloproteinases 1 (TIMP-1), suppressor of cytokine signaling 3 (SOCS3), and interleukin 1 receptor type 2 (IL1R2).

### Patient and clinical characteristics

Twenty-eight patients with sepsis-induced myocardial injury and twenty-five control patients without infections were enrolled in the study. Six of the patients with sepsis-induced myocardial injury died within 30 days. Basic characteristics of patients with sepsis-induced myocardial injury and control patients were comparable (Table 1). Then, we compared the features of patients with sepsis-induced myocardial injury after stratification by 30-day mortality. Basic demographic characteristics, including age, gender, smoking and co-morbidities, were similar between survivors and non-survivors (Table 2). Non-survivors tended to have lower diastolic blood pressure (*P* = 0.05) and higher rates of septic shock (*P* = 0.05) than survivors (Table 3). In addition, non-survivors had increased white blood cell (WBC) count, neutrophil count, troponin I, creatine kinase myocardial band (CK-MB), B-type natriuretic peptide (BNP), sequential organ failure assessment (SOFA) scores, intensive care unit (ICU) admission, and vasopressor use compared with survivors (*P* < 0.01 or *P* < 0.05, Table 3).

**Table 1:** Basic characteristics of patients with and without sepsis-induced myocardial injury.

	**Controls, n = 25**	**Patients, n = 28**	***P* value**
Age (years)	86.2±9.4	88.7±7.9	0.31
Male, n (%)	22 (88.0)	24 (85.7)	1.00
Smoking, n (%)	7 (28.0)	10 (35.7)	0.57
CAD, n (%)	21 (84.0)	23 (82.1)	1.00
Prior MI, n (%)	8 (32.0)	11 (39.3)	0.77
CHF, n (%)	18 (72.0)	24 (85.7)	0.31
Hypertension, n (%)	18 (72.0)	23 (82.1)	0.51
Diabetes, n (%)	8 (32.0)	8 (28.6)	1.00
Dyslipidemia, n (%)	8 (32.0)	9 (32.1)	1.00
COPD, n (%)	10 (40.0)	18 (64.3)	0.10
CKD, n (%)	7 (28.0)	12 (42.9)	0.39
Cirrhosis, n (%)	0 (0)	1 (3.6)	1.00
Malignancy, n (%)	1 (4.0)	4 (14.3)	0.35

Controls refer to subjects without sepsis or infection. Patients refer to patients with sepsis-induced myocardial injury. CAD, coronary artery disease; MI, myocardial infarction; CHF, congestive heart failure; COPD, chronic obstructive pulmonary disease; CKD, chronic kidney disease.

**Table 2:** Basic characteristics of survivors and non-survivor with sepsis-induced myocardial injury.

	**Survivor, n = 22**	**Non-survivor, n = 6**	***P* value**
Age (years)	88.1±8.6	90.7±5.0	0.50
Male, n (%)	19 (86.4)	5 (83.3)	1.00
Smoking, n (%)	9 (40.9)	1 (16.7)	0.37
CAD, n (%)	18 (81.8)	5 (83.3)	1.00
Prior MI, n (%)	7 (31.8)	4 (66.7)	0.17
CHF, n (%)	19 (86.4)	5 (83.3)	1.00
Hypertension, n (%)	17 (77.3)	6 (100)	0.55
Diabetes, n (%)	7 (31.8)	1 (16.7)	0.64
Dyslipidemia, n (%)	8 (36.4)	1 (16.7)	0.63
COPD, n (%)	14 (63.6)	4 (66.7)	1.00
CKD, n (%)	10 (45.5)	2 (33.3)	0.67
Cirrhosis, n (%)	1 (4.5)	0 (0)	1.00
Malignancy, n (%)	2 (9.1)	2 (33.4)	0.19

CAD, coronary artery disease; MI, myocardial infarction; CHF, congestive heart failure; COPD, chronic obstructive pulmonary disease; CKD, chronic kidney disease.

**Table 3:** Clinical characteristics of survivors and non-survivor with sepsis-induced myocardial injury.

**Lung infection, n (%)**	**Survivor, n = 22**	**Non-survivor, n = 6**	***P* value**
	**22 (100)**	**6 (100)**	**1.00**
Heart rate (bpm)	76.6±15.6	81.3±12.5	0.50
SBP (mmHg)	128.5±19.4	124.2±19.0	0.63
DBP (mmHg)	66.5±9.9	58.0±4.0	0.05
Shock, n (%)	2 (9.1)	3 (50.0)	0.05
RR (bpm)	19.1±2.0	20.3±2.9	0.25
PaO_2_ (mmHg)	71.4±27.3	58.6±20.7	0.35
PaCO_2_ (mmHg)	48.1±21.9	41.4±12.8	0.53
WBC (/10^9^L)	7.9±2.9	14.8±7.9	0.00
Neutrophil (/10^9^L)	6.0±2.6	13.1±7.4	0.00
PCT (ng/mL)	2.5±7.8	1.2±1.0	0.69
CRP (mg/L)	30.4±57.4	80.2±93.5	0.12
Troponin I (ng/mL)	0.7±2.4	36.7±63.2	0.00
CK-MB (IU/L)	3.5±3.4	12.8±11.5	0.00
BNP (pg/mL)	322.3±317.8	2614.5±3557.6	0.00
SOFA score	5.8±1.5	8.5±2.5	0.00
ICU admission, n (%)	1 (4.5)	4 (66.7)	0.00
MV, n (%)	1 (4.5)	1 (16.7)	0.39
Vasopressors, n (%)	2 (9.1)	3 (50.0)	0.03

SBP, systolic blood pressure; DBP, diastolic blood pressure; RR, respiratory rate; WBC, white blood cell; PCT, procalcitonin; CRP, C-reactive protein; CK-MB, creatine kinase myocardial band; BNP, B-type natriuretic peptide; SOFA, sequential organ failure assessment; ICU, intensive care unit; MV, mechanical ventilation.

### Circulating eotaxin-1/CCL11 levels in patients with sepsis-induced myocardial injury

**Figure 5:**
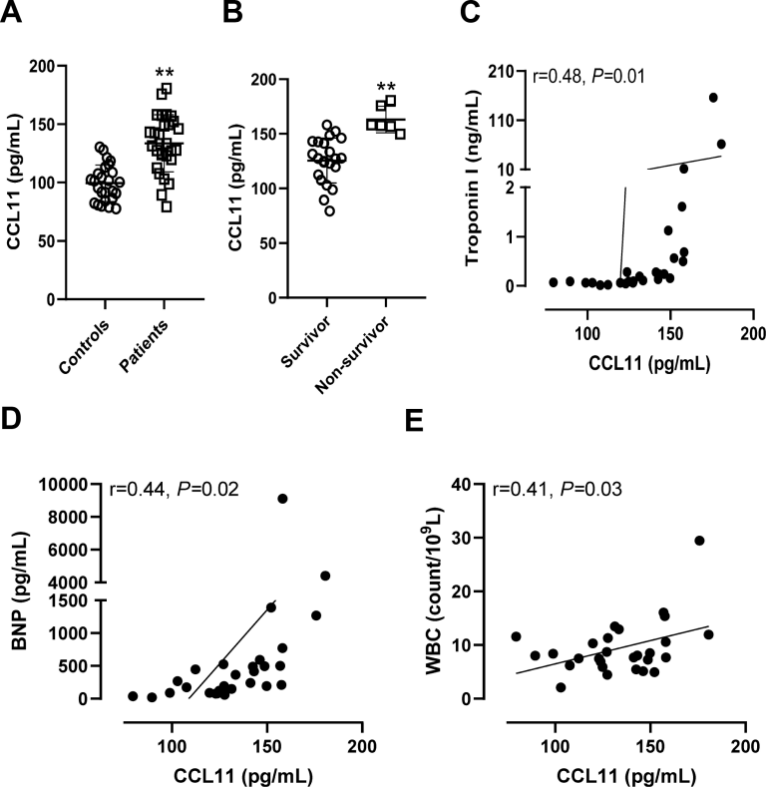
**Circulating CCL11 levels in patients with sepsis-induced myocardial injury.** (**A**) Serum CCL11 levels in control patients without infections and in patients with sepsis-induced myocardial injury. **P < 0.01 *vs.* controls. (**B**) Serum CCL11 levels in survivors and non-survivors with sepsis-induced myocardial injury. **P < 0.01 *vs.* survivors. The correlations between serum CCL11 levels and troponin I (**C**), B-type natriuretic peptide (BNP) (**D**), and white blood cell (WBC) count (**E**).

**Figure 6:**
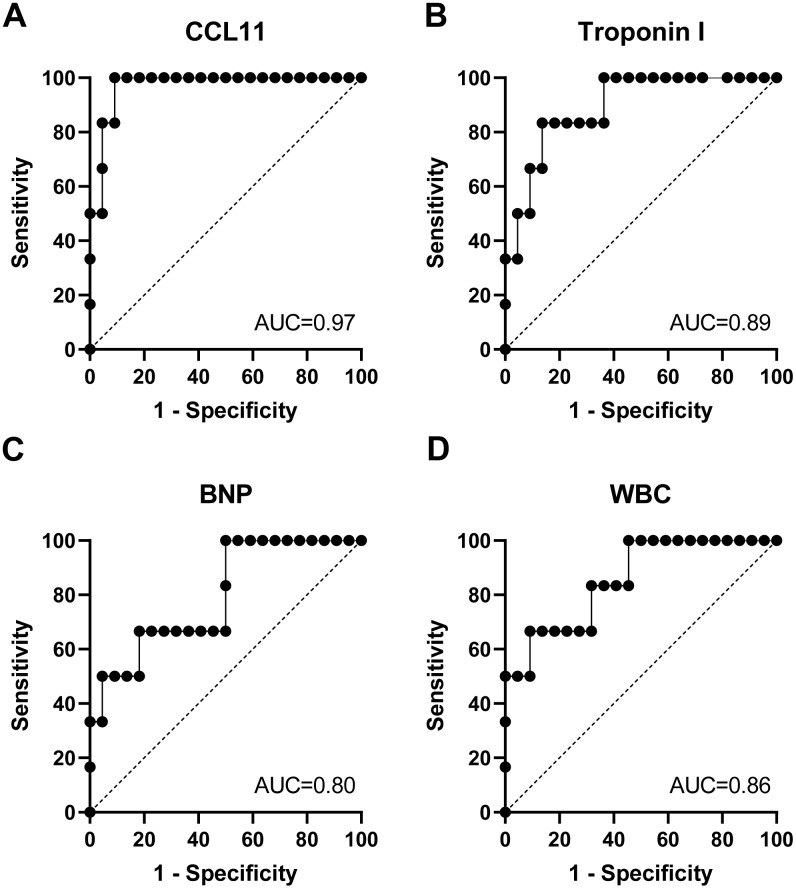
**Circulating CCL11 level is a mortality predictor for elderly patients with septic cardiomyopathy.** The area under the curve (AUC) of receiver operating characteristic (ROC) curves for CCL11 (**A**), troponin I (**B**), B-type natriuretic peptide (BNP) (**C**), and white blood cell (WBC) count (**D**) in predicting death of septic cardiomyopathy.

The circulating eotaxin-1/CCL11 levels were significantly higher in patients with sepsis-induced myocardial injury than control patients without infections (*P* < 0.01, Figure 5A) and were higher in non-survivors than survivors with sepsis-induced myocardial injury (*P* < 0.01, Figure 5B). Serum eotaxin-1/CCL11 levels were positively correlated with troponin I concentrations (r = 0.48, *P* = 0.01, Figure 5C), BNP levels (r = 0.44, *P* = 0.02, Figure 5D), and WBC count (r = 0.41, *P* = 0.03, Figure 5E). As we observed for the prediction of 30-day mortality, eotaxin-1/CCL11 had the greatest discriminatory ability (AUC 0.97, Figure 6A) compared with troponin I concentrations (AUC 0.89, Figure 6B), BNP levels (AUC 0.80, Figure 6C), and WBC count (AUC 0.86, Figure 6D).

## DISCUSSION

In this study, we first identified that eotaxin-1/CCL11 was the most significant DEG in both human and mice with sepsis-induced myocardial injury using bioinformatic analysis. Importantly, we confirmed the role of eotaxin-1/CCL11 in sepsis-induced myocardial injury using the blood samples from elderly patients with sepsis-induced myocardial injury. We found that circulating eotaxin-1/CCL11 levels are positively correlated with the severity of myocardial injury in septic patients and are a good predictor for 30-day mortality.

The definitive diagnostic criteria for septic cardiomyopathy was not available so far and is still a challenge in clinical practice [11]. Currently, ventricular dysfunction evaluated by echocardiography, biochemical markers such as cardiac troponin I and BNP, or hemodynamic markers of inadequate oxygen delivery were used to define septic cardiomyopathy [3, 12]. Elderly patients have high prevalence of pre-existing heart disease with impaired ventricular function and elevated BNP levels. In addition, recent echocardiographic and BNP measurements before they had sepsis were not available in many patients. In our cohort of elderly patients, 85% patients had pre-existing congestive heart failure, so we used increased cardiac troponin I levels to define sepsis-induced myocardial injury in the patients.

Several mechanisms may contribute to pathophysiology of sepsis-induced myocardial injury, including disorganized cytokines, dysregulated nitric oxide synthesis, and mitochondrial dysfunction [13–15]. However, there is still lack of genetic biomarkers that are valuable for evaluating and predicting mortality of septic patients with myocardial injury. Microarray-based transcriptome profile analysis is a powerful tool to identify critical genes involved in pathological processes. Unfortunately, there are only few available microarray datasets of sepsis-induced myocardial injury probably because this disorder is underexamined. We combined the only two datasets of sepsis-induced myocardial injury in human and mice, respectively, and found that 19 Co-DEGs were present in both human and mouse myocardial samples. GO analysis found that these Co-DEGs are primarily involved in inflammation and cytokine-cytokine receptor interaction. Importantly, we identified that eotaxin-1/CCL11 was the most significant up-regulated gene in both human and mice with sepsis-induced myocardial injury. Eotaxin-1/CCL11 is a small glycoprotein that belongs to a family of inflammatory cytokines [16]. Eotaxin-1/CCL11 is primarily involved in recruitment of eosinophils into inflammatory sites and plays a role during allergy-related diseases such as asthma and allergic rhinitis as well as inflammatory disorders such as atherosclerosis [17–19]. The role of eotaxin-1/CCL11 in myocardial disease is largely unknown. Previous studies found that cardiac macrophages and fibroblasts are the main cells producing eotaxin-1/CCL11 that regulates eosinophil recruitment to the heart [20]. It has been reported that eotaxin-1/CCL11 levels were correlated with myocardial fibrosis and mast cell density [21]. However, the role of eotaxin-1/CCL11 in sepsis-induced myocardial injury is still unknown.

The circulating eotaxin-1/CCL11 level has been used as a biomarker of gastric cancer and postmenopausal osteoporosis in clinical trials [22, 23]. The present study demonstrates that serum eotaxin-1/CCL11 level is a potential biomarker that may be valuable for evaluating the severity of myocardial injury in sepsis and predicting mortality. One weakness of this study is that we did not enroll a cohort with sepsis but without myocardial injury. Therefore, the diagnostic value of serum eotaxin-1/CCL11 level in sepsis-induced myocardial injury is still unclear. Although elevation of the circulating eotaxin-1/CCL11 level is associated with the severity and mortality of sepsis-induced myocardial injury, whether eotaxin-1/CCL11 contributes to the development and progression of myocardial injury in sepsis remains unknown. Further animal studies may be helpful to define the pathogenic role of increased eotaxin-1/CCL11 expression in sepsis-induced myocardial injury. Eotaxin-1/CCL11 exerts its function by binding to its specific receptor, C-C motif chemokine receptor 3 (CCR3). Therefore, increased eotaxin-1/CCL11 might induce myocardial injury through enhancing CCR3-mediated inflammation. Once the increased level of eotaxin-1/CCL11 has been proven to be a culprit of sepsis-induced myocardial injury, CCR3 agonists may provide a novel targeted therapy. At present, CCR3 agonists and eotaxin-1/CCL11 neutralizing antibodies are already under development [16]. Therefore, further functional studies of eotaxin-1/CCL11 in sepsis-induced myocardial injury should benefit its treatment. Although eotaxin-1/CCL11 is most abundant in the heart (https://www.proteinatlas.org/ENSG00000172156-CCL11/tissue), it is still not clear whether eotaxin-1/CCL11 is a specific marker for myocardial injury.

A limitation of the present study is that only elderly patients were enrolled. Therefore, it is unclear whether eotaxin-1/CCL11 plays a role in younger patients with sepsis-induced myocardial injury. Eotaxin-1/CCL11 has recently been considered as an aging factor and associated with aging and dementia [24]. Circulating levels of eotaxin-1/CCL11 have been shown to increase with aging in both animals [25] and humans [26] and rise in neurodegenerative diseases [27, 28]. Therefore, it is possible that the elevated eotaxin-1/CCL11 specifically contributed elderly patients with sepsis-induced myocardial injury.

In conclusion, in the present study, we found that eotaxin-1/CCL11 was up-regulated in sepsis-injured myocardium based on bioinformatic analysis and that circulating eotaxin-1/CCL11 was a biomarker for predicting severity and mortality of elderly patients with sepsis-induced myocardial injury based on observational clinical study. These results suggest that eotaxin-1/CCL11 may become a useful biomarkers and potential therapeutic target for sepsis-induced myocardial injury in elderly patients.

## MATERIALS AND METHODS

### Data collection and processing

GSE79962 and GSE53007 datasets were downloaded from GEO (http://www.ncbi.nlm.nih.gov/geo/) [29]. Expression profiling arrays of GSE79962 were generated using GPL6244 [HuGene-1_0-st] Affymetrix Human Gene 1.0 ST Array [transcript (gene) version], while GSE53007 was generated using GPL6885 Illumina MouseRef-8 v2.0 expression beadchip. In GSE79962, 20 left ventricular specimens were obtained from patients with sepsis by rapid autopsy after the patients died and 11 nonfailing left ventricular specimens from brain-dead organ donors were used as controls. In GSE53007, 6 heart muscle specimens from septic mice and 6 normal heart muscle specimens were extracted and analyzed. DEGs between septic cardiomyopathy specimens and control myocardial specimens were identified via GEO2R online tools with log fold change (FC) > 1.2 or log FC < −1.2 and adjust *P* value < 0.05. Then, the Co-DEGs of the GSE79962 and GSE53007 datasets were calculated and illustrated in Venn diagrams. GO functional enrichment, including biological process (BP), molecular function (MF), and cellular component (CC) categories, and KEGG pathway enrichment analyses of the Co-DEGs were performed using the Database for Annotation, Visualization and Integrated Discovery (DAVID), an online bioinformatic tool. GO terms and KEGG pathways of biological functions with *P* values less than 0.05 was considered to be significantly enriched. Protein–protein interaction (PPI) networks of Co-DEGs were analyzed using the search tool for the retrieval of interacting genes (STRING database, V10.5; http://string-db.org/) which predicts functional associations of proteins and protein-protein interactions. Then, we saved and exported an interaction network chart with a combined score > 0.4. Nodes with high centrality in the protein interaction network were considered to have an essential role in the network.

### Patients and study design

This is a prospective observational study conducted from January 2019 to August 2019 in the Department of Geriatrics of a tertiary general hospital. Informed consent was obtained from all patients. The Institutional Review Board of the General Hospital of Western Theater Command reviewed and approved the study protocol. The Department of Geriatrics is a specialized unit particularly for elderly veterans. All patients with the diagnosis of sepsis and myocardial injury were included. Two patients who were under the age of eighty were excluded. No patients had acute coronary syndrome or cardiac surgery in the last two weeks. In total, 28 patients with sepsis and myocardial injury were enrolled in the study. Sepsis was defined according to The Third International Consensus Definitions for Sepsis and Septic Shock [30]. Myocardial injury was defined as cardiac troponin I > 3-fold the normal upper limit without symptoms or signs of acute coronary syndrome (no chest pain or ST segment elevation or depression). Twenty-five age- and sex-matched patients without infections were used as controls.

### Clinical and laboratory data

Demographic and clinical data, including age, sex, smoking, co-morbidities, vital signs, intensive care unit admission, mechanical ventilation, and use of vasopressors were collected. Complete blood count, blood procalcitonin, C-reactive protein, cardiac Troponin I, CK-MB, BNP, and arterial blood gas analysis were performed in the first full day after admission. The SOFA scores were calculated [30]. Follow-up data included 30-day mortality rate. Peripheral blood samples were collected, and the serum was separated and stored at -80 °C freezer until measurement of serum C-C motif chemokine 11 (CCL11) by enzyme-linked immunosorbent assay (R&D Systems, Minneapolis, MN, USA).

### Statistics

Data are presented as mean and standard deviation for continuous variables and as number and percentage of patients for categoric variables. Comparing means of continuous variables between two groups were performed using the *t*-test, and categorical data were analyzed using the chi-square test. Pearson correlation and linear regression were performed to examine the relationship between two parameters. The area under the curve (AUC) of receiver operating characteristic (ROC) curves were calculated using Prism GraphPad. Differences were considered significant at a probability level of *P* < 0.05. Statistical analysis was performed with SSPS version 21.0 software (IBM Corp., Armonk, NY, USA).
